# Gender difference in socioeconomic factors affecting suicidal ideation and suicidal attempts among community-dwelling elderly: based on the Korea Community Health Survey

**DOI:** 10.4178/epih.e2020052

**Published:** 2020-07-13

**Authors:** Jin-Young Jeong

**Affiliations:** Research Institute of Clinical Epidemiology, Hallym University, Chuncheon, Korea

**Keywords:** Suicide, Socioeconomic factors, Aged, Community Health Survey

## Abstract

**OBJECTIVES:**

This study was performed to explore socioeconomic factors associated with suicidal ideation and suicidal attempts among the local community’s resident elderly.

**METHODS:**

The subjects included 129,277 participants aged 65 years or above of the Korea Community Health Survey conducted in 2013 and 2017. Based on the questions for suicidal ideation and suicidal attempts, the subjects were divided into a no suicidal ideation group (n=111,344), a suicidal ideation group (n=17,487), and a suicidal attempt group (n=446). All analyses were stratified by gender, and a complex sample logistic regression analysis was performed to analyze associated factors. SAS version 9.4 was used for all analyses with a significance level of 0.05.

**RESULTS:**

Common factors associated with suicidal ideation in both genders included marital status, frequency of contact with friends, social activity, and average monthly household income. Economic activity was demonstrated as relevant only to the elderly men subjects. According to the analysis, factors associated with suicidal attempts were the recipients of the National Basic Living Security Act for the elderly men compared to age, frequency of contact with family, frequency of contact with friends, and average monthly household income for the elderly women.

**CONCLUSIONS:**

The study revealed that socioeconomic factors leading to suicidal ideation were similar in the elderly of both genders, while a difference was demonstrated for factors associated with suicidal attempts between the elderly of both genders. It is expected that the results of this study may be used as the basis for screening the local community’s elderly with a high suicidal risk, and in the development of suicide prevention services.

## INTRODUCTION

The incidence of suicidal deaths among the Korean elderly was 48.6 cases per 100,000 population in 2018 and has been steadily decreasing since peaking at 81.9 cases per 100,000 population in 2010 [1]. However, one in four people who die by suicide is an elderly, and the incidence of suicidal deaths among the elderly is 1.8 times higher than that among the entire Korean population at 26.6 cases per 100,000 population.

Korea has fully transitioned into an aged society with the elderly accounting for over 14% of the entire Korean population in 2018 [2]. While an aged society is the positive result of medical advances and improvements in living conditions cumulatively leading to an average life expectancy, being an aged society has negative outcomes such as a high rate of elderly poverty close to 50% [3], an extended period of living with a disease, and the deterioration of family support systems due to family nuclearization.

The biggest limitation in suicide research is that people who have committed suicide cannot be directly included in the research process. Research on suicidal attempt can more closely examine the causes of suicide compared to research on suicidal ideation since suicidal attempt is closer to a suicidal death compared to suicidal ideation. However, previous studies on suicidal attempts are mostly qualitative studies on a small number of suicidal attempters [4-7], and extremely few quantitative studies have been conducted on suicidal attempts likely due to the difficulty in gathering participants who have attempted suicide [8-10]. In the 2017 Korean National Survey on the Elderly [11], which is a major survey on the elderly, only 0.9% (n= 89) of all survey participants attempted suicide. The scarcity of suicidal attempters limits the research on suicidal attempts. To identify high-risk groups, provide interventions, and obtain generalized results based on monitoring, a large-scale quantitative study involving a general population group is necessary.

While socioeconomic factors have a significant influence on the quality of life of the elderly, demographic and socioeconomic factors have been used as control variables rather than being examined as a major topic of research in domestic studies on suicide among the elderly [12-14]. Women are reported to have a higher rate of suicidal ideation, while men have a higher rate of attempting or committing suicide [1,11,15], indicating gender differences in terms of suicide.

In this study, the Korea Community Health Survey (CHS), a large-scale epidemiological data set representing the Korean population, was used to examine the factors associated with suicidal behavior among the elderly in local communities after stratifying the data by gender. The objectives of this study are to calculate the rate of suicidal ideation and the rate of suicidal attempts in different gender and age groups, and to analyze the associations of suicidal ideation and suicidal attempts with demographic and economic factors as well as social relationships while controlling the effect of physical and psychological health factors.

## MATERIALS AND METHODS

### Participants

The CHS includes a four-year rolling survey that asks suicide-related questions and has been conducted a total of three times (2009, 2013, 2017) in all regions across the country. In this study, the CHS data from 2013 and 2017 were used. The rate of suicide among the elderly started to decrease in 2010. Since it was deemed that the results regarding suicide-related factors could differ between 2009, during which the rate of suicide increased, and the years thereafter, the data from 2009 were not included.

After excluding 65 respondents who did not respond to the questions regarding suicidal ideation and suicidal attempt, and 45 respondents who responded “no” to having had suicidal ideation but “yes” to having made a suicidal attempt, a total of 129,277 respondents aged 65 years or older were included in this study.

### Data collection

The CHS was introduced in 2008 to establish regional healthcare plans and obtain health statistics from cities/counties/districts to assess the performance of regional healthcare projects [16]. The target participants are adults aged 19 years or older with a sample size of 900 persons (target error ± 3%) per city/county/district. Approximately 230,000 adults are surveyed per year [17]. Using resident registration address data as a sampling frame, primary sampling locations and secondary sampling households are selected by probability-proportional-to-size sampling and systematic sampling, respectively. An investigator visits each household to survey participants by means of computer-assisted personal interviewing using a laptop. The survey investigates the type of generation, household income, health behaviors, vaccination and health examination, disease contraction, use of medical services, accidents and addiction, limited daily activities, quality of life, use of healthcare facilities, socio-physical environment, personal hygiene, women health, education, and economic activities.

### Variables

#### Dependent variables

The dependent variables used in this study are suicidal ideation and suicidal attempt. Each variable had a single question. Participants answered either “yes” or “no” to the following questions for suicidal ideation and suicidal attempt, respectively: “Have you wanted to commit suicide in the last one year?” and “Have you actually attempted committing suicide in the last one year?” Since the objective of this study is to identify the factors affecting suicidal ideation and suicidal attempt, the participants were divided into a no-suicidal-ideation group (n= 111,344), a suicidal ideation group (n= 17,487), and a suicidal attempt group (n= 446) based on their responses to the questions on suicidal ideation and suicidal attempt.

#### Independent variables

The following independent variables were included in this study: demographic factors (age, marital status, living alone), economic factors (monthly household income, recipient of the Basic Livelihood Security, economic activity), and social networks (frequency of contact with family/friends/neighbors, social activity).

The participants were either aged 65-74 years or ≤ 75 years. Marital status was classified as “living together with a partner,” “widowed,” or “separated/divorced/single.” A participant was deemed as “living alone” if he lived in a single-person household. The average monthly household income investigated in 2013 was an open type, and that in 2017 was a closed type. There were four categories of average monthly household income: < 500,000, 500,000-990,000, 1,000,000-1,990,000, and ≥ 2,000,000 Korean won (KRW). The participants were classified as either “a current or former recipient of the National Basic Living Security Act” or “not a recipient of the National Basic Living Security Act.” The frequencies of contact with relatives (including family), neighbors, and friends were “less than once per month,” “1-3 times per month,” and “more than once per month.” A participant was deemed to have social activities if he participated in at least one of the following activities at a minimum of once per month: religious, socializing, recreation/leisure, or volunteer activity.

#### Control variables

Psychological health (depression, stress), physical function (fall, being bedridden, accident/addiction, limited daily activities), quality of life (subjective health status, difficulty chewing, pain/discomfort), and use of medical services (not receiving necessary medical services), all of which are known to affect suicidal ideation and suicidal attempt, were included as the control variables.

### Statistical analysis

The CHS sample populations are derived from a sampling design with cities/counties/districts as sampling units, and weights based on the structure of the sampling design are applied [17]. All analyses were performed using complex samples considering the research design of the CHS.

The data for each variable were presented as the number of participants (%) and the weighted sampling error (%) (Table 1). The demographic and social characteristics of the no suicidal ideation, suicidal ideation, and suicidal attempt groups in Table 2 were compared in a cross-tabulation analysis.

A logistic regression analysis was performed to analyze the factors associated with suicidal ideation and suicidal attempt (Table 3). The factors associated with suicidal ideation were identified based on the analysis results of the no suicidal ideation and suicidal ideation groups. The factors associated with suicidal attempt were identified based on the analysis results of the suicidal ideation and suicidal attempt groups.

A multivariate logistic regression analysis was performed after adjusting for the effect of psychological health (depression, stress), physical function (fall, being bedridden, accident/addiction, limited daily activities), quality of life (subjective health status, difficulty chewing, pain/discomfort), and not receiving necessary medical services. In all analyses, the participants were stratified by gender for gender comparison. All analyses were performed using SAS version 9.4 (SAS Institute Inc., Cary, NC, USA) with the level of significance at 0.05.

### Ethics statement

The CHS was approved by the Institutional Review Board (IRB) of the Korea Centers for Disease Control and Prevention. The approval number in 2013 was 06EXP-01-3C. The CHS was categorized as research involving humans based on Article 2, Clause 2 of the Bioethics and Safety Act, and was excluded from the IRB review after 2017. The raw CHS data were downloaded from the CHS website after applying for and receiving permission to download from the administrator of the CHS website (January 2019, 2020).

## RESULTS

A total of 129,277 participants were included in this study. Of these, 41.2% were men, and 58.8% were women (Table 1). Further, 39.7% and 46.2 % of the men and women participants, respectively, were aged ≥ 75 years; women were generally older than men, and 87.2% and 45.4% of the men and women participants, respectively, had a spouse. Moreover, 10.2% and 34.5% of the men and women participants, respectively, lived alone. Both genders had the highest frequency of contact for neighbors, followed by family (relatives), and friends. Additionally, 43.0% and 56.5% of the men and women participants, respectively, had an average monthly household income of < 1,000,000 KRW. Further, 5.5% and 8.5% of the men and women participants, respectively, were current or former recipients of the National Basic Living Security Act; women were generally in a poorer financial situation.

**Figure 1.**
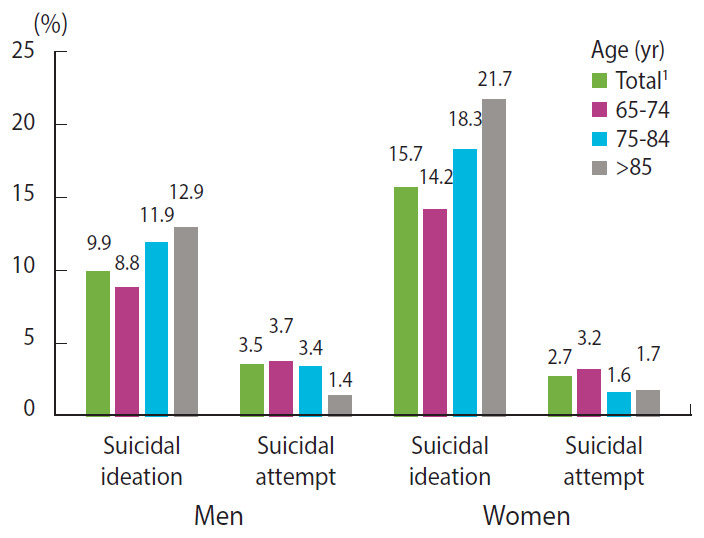
Suicidal ideation and suicidal attempt rates (%) by gender and age groups. ^1^Age standardization with 2005 population.

The rate of suicidal ideation was 1.6 times higher for women (15.7%) than men (9.9%) (Figure 1). The rate of suicidal ideation increased with age for both genders. The ratio between the rates of suicidal ideation of the two genders (women/men) was consistent for all age categories: 1.61 for 65-74 years, 1.54 for 75-84 years, and 1.68 for ≥ 85 years. The rate of suicidal attempt was 1.3 times higher for men (3.5%) than women (2.7%). The rate of suicidal attempt decreased after the age of 85 for men and 75 for women.

The demographic and social characteristics of the suicidal ideation and suicidal attempt groups are presented in Table 2. Differences were found for all factors except for age in men, and social and economic activities in women. While the ratio between the participants aged 65-74 years to those aged ≥ 75 years was 6:4 in both groups for men, the ratio significantly differed between the two groups (5:5 and 7:3, respectively) for women. A high percentage of the participants in the suicidal ideation group lived with a spouse, and a high percentage of participants in the suicidal attempt group were divorced/single/separated for both genders. A high percentage of the men and women participants in the suicidal attempt group also lived alone. With respect to the social factors, a low percentage of the participants in the suicidal attempt group were in contact with their family (relatives), neighbors, and friends at least once per week and had social activities, indicating that the suicidal attempt group had a smaller social network compared to the other groups. With respect to the economic factors, a high percentage of the participants in the suicidal attempt group had an average monthly household income of < 500,000 KRW and were current or former recipients of the National Basic Living Security Act. The p-values presented in Table 2 represent the differences between the suicidal ideation and suicidal attempt groups.

The factors associated with suicidal ideation and suicidal attempt were analyzed after adjusting for the control variables. The results of the analysis are presented in Table 3. For men, marital status, average monthly household income, frequency of contact with friends, and social activities were identified as the factors associated with suicidal ideation and suicidal attempt. For women, marital status, monthly household income, economic activities, frequency of contact with friends, and social activities were associated with suicidal ideation and suicidal attempt. The factors associated with suicidal attempt were age, monthly household income, and frequencies of contact with family (relative) and friends.

## DISCUSSION

In this study, no unusual trends in the rate of suicidal ideation and suicidal attempt were found across all ages for men, while the rate of suicidal attempt was lower for women aged ≥ 75 years compared to those aged 65-74 years. Doh & Hoe [18], who examined suicide-related factors using data from the 6th Korean Welfare Panel, reported low age, high levels of depression, living alone, and low satisfaction with family relationships to be associated with suicidal attempt. The high rate of suicidal attempt among the young elderly has been attributed to the increase in the risk of suicide among the young elderly who have just been categorized as being elderly, and there is a possibility that the rate of suicidal attempt among the elderly aged ≥ 75 years was underestimated since older individuals tend to avoid talking about suicide [19].

Unlike the rate of suicidal attempt, the rate of suicidal death increased with age in this study. The rate of suicidal death was 58.8, 117.4, and 140.9 cases per 100,000 population for men aged 65-74 years, 75-84 years, and ≥ 85 years, respectively, and 16.0, 28.5, and 42.5 cases per 100,000 population for women with respect to the above years. For both genders, the rate of suicidal attempt decreased, and the rate of suicidal death increased with age. This result may be attributed to the high rate of successful suicidal attempts among the elderly. The elderly are reported to have high rates of successful suicidal attempts because they are less likely to inform others of their suicidal intent and use more fatal methods of committing suicide compared to the younger age groups [20]. While the rate of suicidal attempt (3.5% for men and 2.7% for women) did not significantly differ between the two genders, the rate of suicidal death was 3.5 times higher for men than women, suggesting that men have a higher rate of successfully committing suicide than women. According to the 2018 National Survey on Suicide, a higher percentage of men were well-prepared to commit suicide with a detailed suicidal plan and executed their plan compared to women [21]. Although men have lower rates of suicidal attempt, elderly men of advanced age are a high-risk group with a high rate of successful suicidal attempts and thus require great care.

A suicidal attempt is a strong predictor of additional suicidal attempts, and repeated suicidal attempts increase the likelihood of a suicidal death. Thus, it is important to pay attention to men and young elderly who have higher rates of suicidal attempts compared to women.

Conflicting results have been reported regarding the association between marital status and suicidal attempt among the elderly. Some studies have reported that marriage prevents suicidal attempt [22,23], while others have reported no association between marriage and suicidal attempt [10,24]. However, the results of these studies cannot be directly compared to those of the present study since the studies did not stratify their data by gender. The death of a spouse is one of the most negative life experiences. Bereavement was significantly less likely to happen in men (8.7%) than women (52.1%) (Table 1). Therefore, men may be more likely to experience the shock and sense of loss from bereavement and to have suicidal ideation compared to women. Furthermore, the lack of skills necessary for independent living such as house chore and cooking skills in elderly men may have contributed to their high rate of suicidal ideation. However, bereavement did not affect the rate of suicidal attempt in both elderly men and women. It has been reported that 50.4% of the elderly have at least two chronic diseases [12], and the top five causes of death in the elderly (cancer, heart disease, cerebrovascular disease, pneumonia, and diabetes) account for 57.2% of the total deaths in the elderly [1]. Assuming that bereavement at old age occurs in the order of diagnosis of a disease, treatment, disease worsening, and death, it is possible that the elderly mentally prepare themselves for the death of their spouses during this process, and their suicidal ideation from the sense of loss does not lead to a suicidal attempt owing to the mental preparation.

Social networks may also mitigate the risk of suicidal ideation and suicidal attempt. Social support systems are known to protect individuals from suicidal attempts [10,25-28]. A spouse or a family that lives with an elderly individual not only helps him or her with his or her daily life but also provides emotional support to prevent the deterioration of emotional health and suicidal behaviors.

In this study, a significantly high percentage of elderly women attempted suicide due to lack of emotional support. The elderly who were in contact with their families (relatives) less than once per month and their friends less than 1-3 times per month also had a high-risk of suicidal attempt. Families provide emotional and financial support; therefore, the frequency of contact with family can be a major predictor of suicidal attempt. While the elderly who were in contact with their friends 1-3 times per month had a high-risk of suicidal attempt, those who were in contact with friends less than once per month did not. Friends that one is in contact with less than once per month are not likely close friends from whom one may expect to receive emotional support. In previous studies, the risk of suicide decreased as the number of people whom one was close to or received help from increased [13] and as one engaged in more social activities [10].

In summary, the risk of suicidal ideation increases when the closest support system collapses, and the risk of suicidal attempt increases when other external support systems that work as a buffer collapse. In this study, social support was measured by examining the marital status and the frequency of contact with those in a personal social network; the stability of support systems was not examined. This stability must be assessed by measuring the intimacy of relationships and the quantity and quality of social contact. Since the demand for public support for the elderly has increased due to the increase in the number of single-person households and changes in family relationships, it is also necessary to assess the contributions of public social networks including social workers and home-care nurses in elderly care.

The economic factors associated with suicidal attempt were “being a current or former recipient of the National Basic Living Security Act” for men, and “average monthly household income of < 1,000,000 KRW” for women. While both are economic factors, “current or former recipient of the National Basic Living Security Act” was indicative of relative poverty while a “monthly household income of < 1,000,000 KRW” was indicative of absolute poverty.

The recipients of the National Basic Living Security Act accounted for 3.6% (n= 1,881,357) of the total Korean population in 2019 [29]. Traditionally, men have been the breadwinners of the family with clear gender roles. Being a recipient of the National Basic Living Security Act means that one’s family is one of the poorest families in the country. Upon losing their financial abilities, men can consider themselves as no longer useful. In a study that examined the predictors of suicidal ideation in elderly men of varying ages, being a recipient of the National Basic Living Security Act was a significant predictor of suicidal attempt among the old-old elderly (65-74 years) [30]. Elderly women have placed higher importance on being financially independent, which may be why average monthly household income, an indicator of the absolute financial status, affected the rate of suicidal attempts in the elderly women.

The relative poverty rate of the elderly (current income ≤ 50% of the median income) was 49.4% in the second of the four quarters in 2018, and one in two elderly people was impoverished [3]. The basic pension plan introduced in July 2014 to elderly poverty provided financial support for 70% of the total elderly population and exhibits the nature of a universal social security system. Therefore, elderly men who have negative views about public assistance may have a less negative view on being a recipient of basic pensions. The basic pension plan has led to an overall increase in income, mitigated income inequality, and has had a psychological effect on its recipients [15]. While providing and increasing a basic pension may help reduce elderly suicide, it may not bring the elderly with low income above the poverty line. In addition to introducing income policies aimed at creating more job opportunities for the elderly and increasing their basic pensions, effort to provide psychological support to reduce suicidal behaviors caused by financial difficulties is needed. The elderly who do not receive sufficient social support on their own may benefit from social support from communities.

This study has several limitations. First, as a cross-sectional study, it does not reveal any causal relationships between demographic and social factors and suicidal attempt in the elderly. Second, there is a possibility that the elderly who used relatively less fatal methods of committing suicide and are now living a relatively healthy life in a local community were selectively included in this study. Third, although suicide is known to occur in the order of suicidal ideation, suicidal plan, and suicidal attempt, the factors associated with suicidal plan were not included in this study since the CHS does not collect any information about suicidal plan. In addition, the question on the CHS asking whether the participants had ever wanted to commit suicide may not directly investigate whether the participants actually had suicidal ideation. Furthermore, an in-depth analysis of the factors related to suicidal attempt could not be performed since the suicidal attempt survey used in this study did not include factors such as the method, frequency, and fatality of suicidal attempt. Fourth, even after combining the data from two separate years to include a sufficient number of suicidal attempters, only 446 suicidal attempters (183 men and 263 women) were included. The multivariate analysis performed in this study may lack statistical power for certain variables that had a small number of participants after stratification by gender. Fifth, there is a possibility that different factors may be found to be associated with suicidal ideation and suicidal attempt between 2013 and 2017, and that combining the data from these two years reduced the significance of the associations that certain factors had with suicidal ideation and suicidal attempt. Since the National Basic Living Security Act was introduced in 2014, there is a high possibility that different economic factors were associated with suicide between 2013 and 2017. An additional study on the impact of being a recipient of the National Basic Living Security Act on the elderly, particularly, the impoverished elderly, is necessary.

This study analyzed the association of suicidal attempt with demographic and social factors. Differences in terms of age groups, economic factors, and types of social networks were found between the two genders. Based on these results, the impoverished elderly or the elderly who have weak social support may be identified as a high-risk group in terms of suicidal attempt. A more systematic follow-up observation of these elderly populations is suggested.

## Figures and Tables

**Table 1.** Socio-demographic characteristics of the subjects

Variables		Categories	n (%)		W% (SE)	
			Men (n=53,239)	Women (n=76,038)	Men (N=5,920,335)	Women (N=7,715,323)
Demographic factors						
	Age (yr)	65-74	32,109 (60.3)	40,904 (53.8)	63.8 (0.30)	58.4 (0.27)
		≥75	21,130 (39.7)	35,134 (46.2)	36.2 (0.30)	41.6 (0.27)
	Marital status	Living together	46,318 (87.2)	34,392 (45.4)	87.2 (0.22)	46.7 (0.27)
		Bereaved	4,623 (8.7)	39,525 (52.1)	8.1 (0.17)	49.5 (0.27)
		Divorced/single/separated	2,159 (4.1)	1,889 (2.5)	4.7 (0.14)	3.7 (0.11)
	Living alone	No	47,795 (89.8)	49,786 (65.5)	91.2 (0.17)	73.3 (0.23)
		Yes	5,441 (10.2)	26,248 (34.5)	8.8 (0.17)	26.7 (0.23)
Social network						
	Frequency of family contact	≥1/wk	29,963 (56.3)	47,406 (62.4)	50.8 (0.33)	58.6 (0.29)
		1-3 times/mo	8,121 (15.3)	10,317 (13.6)	16.1 (0.24)	14.2 (0.19)
		<1/mo	15,124 (28.4)	18,278 (24.0)	33.1 (0.32)	27.2 (0.26)
	Frequency of neighborhood contact	≥1/wk	39,021 (73.4)	61,453 (80.9)	56.8 (0.33)	68.6 (0.27)
		1-3 times/mo	2,683 (5.1)	3,104 (4.1)	6.3 (0.16)	5.2 (0.13)
		<1/mo	11,431 (21.5)	11,377 (15.0)	36.9 (0.33)	26.2 (0.26)
	Frequency of friend contact	≥1/wk	24,993 (47.0)	33,960 (44.8)	45.8 (0.32)	45.8 (0.28)
		1-3 times/mo	6,251 (11.8)	5,677 (7.5)	12.6 (0.21)	8.4 (0.16)
		<1/mo	21,899 (41.2)	36,119 (47.7)	41.6 (0.32)	45.8 (0.28)
	Social activities	Yes	34,954 (65.7)	46,570 (61.3)	69.2 (0.29)	67.5 (0.25)
		No	18,282 (34.3)	29,459 (38.7)	30.8 (0.29)	32.5 (0.25)
Economic factors						
	Household income (10^4^ Korean won/mo)	≥200	15,269 (29.3)	17,687 (23.8)	38.9 (0.33)	34.1 (0.28)
		100-199	14,449 (27.7)	14,695 (19.7)	27.7 (0.28)	21.6 (0.23)
		50-99	14,845 (28.4)	21,763 (29.3)	23.4 (0.26)	21.6 (0.23)
		<50	7,636 (14.6)	20,274 (27.2)	10.0 (0.17)	18.5 (0.20)
	Recipients of National Basic Living Security Act	None	50,316 (94.5)	69,535 (91.5)	94.2 (0.16)	91.6 (0.17)
		Ex- or current recipient	2,917 (5.5)	6,471 (8.5)	5.8 (0.16)	8.4 (0.17)
	Economic activity	Yes	26,240 (49.3)	23,875 (31.4)	38.8 (0.30)	20.6 (0.20)
		No	26,994 (50.7)	52,156 (68.6)	61.2 (0.30)	79.4 (0.20)

n, unweighted sample size; N, weighted sample size; W, weighted; SE, standard error; KRW, Korean won.

**Table 2.** Characteristics of suicidal ideation and suicidal attempt group by gender

Variables		Categories	Men			Women		
			No suicidal ideation (n=47,794)	Suicidal ideation (n=5,262)	Suicidal attempt (n=183)	No suicidal ideation (n=63,550)	Suicidal ideation (n=12,225)	Suicidal attempt (n=263)
Demographic factors								
	Age (yr)	65-74	64.6 (0.31)	56.2 (1.00)	60.4 (5.72)	59.8 (0.29)	50.9 (0.68)	68.2 (3.66)
		≥75	35.4 (0.31)	43.8 (1.00)	39.6 (5.72)	40.2 (0.29)	49.1 (0.68)	31.8 (3.66)
				0.151			<0.001	
	Marital status	Living together	88.4 (0.22)	76.6 (0.87)	68.6 (5.10)	47.8 (0.30)	41.2 (0.68)	37.1 (3.90)
		Bereaved	7.3 (0.17)	15.5 (0.75)	14.4 (3.21)	48.6 (0.30)	54.6 (0.70)	51.7 (4.14)
		Divorced/single/separated	4.3 (0.14)	7.9 (0.55)	17.0 (4.01)	3.6 (0.12)	4.2 (0.28)	11.2 (2.84)
		p-value		<0.001			<0.001	
	Living alone	No	92.0 (0.17)	83.4 (0.71)	71.6 (4.81)	74.2 (0.24)	69.2 (0.59)	59.9 (4.07)
		Yes	8.0 (0.17)	16.6 (0.71)	28.4 (4.81)	25.8 (0.24)	30.8 (0.59)	40.1 (4.07)
		p-value		<0.001			<0.001	
Social network								
	Frequency of family contact	≥1/wk	51.5 (0.33)	44.5 (1.00)	41.8 (5.63)	59.5 (0.31)	54.3 (0.68)	35.3 (3.76)
		1-3 times/mo	16.2 (0.25)	15.0 (0.71)	10.3 (3.18)	14.2 (0.21)	14.2 (0.48)	12.1 (2.52)
		<1/mo	32.3 (0.32)	40.5 (1.00)	47.9 (5.56)	26.3 (0.28)	31.5 (0.65)	52.6 (4.06)
		p-value		0.003			<0.001	
	Frequency of neighborhood contact	≥1/wk	57.2 (0.33)	53.1 (1.02)	49.9 (5.58)	69.2 (0.29)	65.7 (0.69)	57.1 (4.26)
		1-3 times /mo	6.5 (0.17)	4.9 (0.41)	11.2 (5.69)	5.3 (0.14)	4.8 (0.29)	3.7 (1.55)
		<1/mo	36.3 (0.34)	42.0 (1.02)	38.9 (5.37)	25.5 (0.28)	29.5 (0.68)	39.2 (4.29)
		p-value		<0.001			0.007	
	Frequency of friend contact	≥1/wk	47.1 (0.33)	34.0 (0.95)	28.3 (4.65)	47.3 (0.30)	38.2 (0.68)	30.1 (3.56)
		1-3 times/mo	12.9 (0.22)	9.7 (0.61)	8.5 (3.43)	8.7 (0.17)	7.1 (0.37)	8.9 (2.72)
		<1/mo	40.0 (0.32)	56.3 (1.00)	63.2 (5.29)	44.0 (0.30)	54.7 (0.69)	61.0 (3.96)
		p-value		0.022			0.029	
	Social activities	Yes	71.2 (0.29)	51.8 (1.02)	43.8 (5.57)	69.9 (0.27)	55.2 (0.67)	50.2 (4.15)
		No	28.8 (0.29)	48.2 (1.02)	56.2 (5.57)	30.1 (0.27)	44.8 (0.67)	49.8 (4.15)
		p-value		0.006			0.125	
Economic factors								
	Household income (104 Korean won/mo)	≥200	40.4 (0.33)	26.0 (0.96)	14.2 (5.81)	35.8 (0.31)	25.6 (0.65)	16.4 (3.31)
		100-199	28.2 (0.30)	22.6 (0.84)	19.6 (4.23)	22.2 (0.25)	18.9 (0.55)	13.5 (2.70)
		50-99	22.6 (0.27)	30.5 (0.93)	33.2 (5.29)	25.3 (0.25)	28.0 (0.61)	29.8 (3.81)
		<50	8.8 (0.16)	20.9 (0.76)	32.9 (5.19)	16.7 (0.20)	27.6 (0.56)	40.3 (4.11)
		p-value		<0.001			<0.001	
	Recipients of National Basic Living Security Act	None	95.0 (0.15)	87.3 (0.68)	74.4 (4.74)	92.8 (0.16)	85.5 (0.49)	77.4 (3.70)
		Ex -or current recipient	5.0 (0.15)	12.7 (0.68)	25.6 (4.74)	7.2 (0.17)	14.5 (0.49)	22.6 (3.70)
		p-value		<0.001			0.002	
	Economic activity	Yes	40.2 (0.32)	26.6 (0.83)	19.0 (3.73)	21.4 (0.22)	16.7 (0.46)	16.5 (2.82)
		No	59.8 (0.32)	73.4 (0.83)	81.0 (3.73)	78.6 (0.22)	83.3 (0.46)	83.5 (2.82)
		p-value		0.001			0.92	

Values are presented as % (standard error). p-value between suicidal ideation and suicidal attempt.

**Table 3.** Related-factors of Suicidal ideation and suicidal attempt in elderly Koreans^1^

Variables		Categories	Men		Women	
			Suicidal ideation	Suicidal attempt	Suicidal ideation	Suicidal attempt
Demographic factors						
	Age (yr)	65-74	1.00 (reference)	1.00 (reference)	1.00 (reference)	1.00 (reference)
		≥75	0.95 (0.85, 1.05)	0.91 (0.56, 1.48)	1.04 (0.96, 1.12)	0.52 (0.37, 0.73)
	Marital status	Living together	1.00 (reference)	1.00 (reference)	1.00 (reference)	1.00 (reference)
		Bereaved	1.73 (1.37, 2.18)	0.49 (0.24, 1.04)	1.17 (1.06, 1.29)	1.15 (0.73, 1.81)
		Divorced/single/separated	1.20 (0.90, 1.60)	0.89 (0.44, 1.77)	1.15 (0.93, 1.43)	1.57 (0.87, 2.82)
	Living alone	No	1.00 (reference)	1.00 (reference)	1.00 (reference)	1.00 (reference)
		Yes	0.99 (0.77, 1.28)	1.87 (0.95, 3.67)	0.93 (0.83, 1.04)	1.09 (0.69, 1.71)
Social network						
	Frequency of family contact	≥1/wk	1.00 (reference)	1.00 (reference)	1.00 (reference)	1.00 (reference)
		1-3 times /mo	1.04 (0.91, 1.20)	0.83 (0.47, 1.45)	1.02 (0.92, 1.13)	1.24 (0.84, 1.84)
		<1/mo	1.07 (0.96, 1.20)	1.00 (0.63, 1.60)	0.99 (0.91, 1.07)	2.12 (1.55, 2.90)
	Frequency of neighborhood contact	≥1/wk	1.00 (reference)	1.00 (reference)	1.00 (reference)	1.00 (reference)
		1-3 times/mo	0.76 (0.62, 1.94)	1.99 (0.79, 5.01)	0.87 (0.74, 1.02)	0.60 (0.25, 1.46)
		<1/mo	0.94 (0.83, 1.05)	0.66 (0.43, 1.01)	1.04 (0.95, 1.13)	1.12 (0.81, 1.53)
	Frequency of friend contact	≥1/wk	1.00 (reference)	1.00 (reference)	1.00 (reference)	1.00 (reference)
		1-3 times/mo	1.02 (0.85, 1.21)	1.18 (0.56, 2.49)	1.09 (0.94, 1.25)	1.80 (1.03, 3.13)
		<1/mo	1.17 (1.05, 1.31)	1.29 (0.84, 1.98)	1.09 (1.01, 1.17)	1.16 (0.86, 1.57)
	Social activities	Yes	1.00 (reference)	1.00 (reference)	1.00 (reference)	1.00 (reference)
		No	1.26 (1.14, 1.40)	1.10 (0.73, 1.66)	1.32 (1.22, 1.41)	1.24 (0.91, 1.68)
Economic factors						
	Household income (10^4^ Korean won/mo)	≥200	100 (reference)	1.00 (reference)	1.00 (reference)	1.00 (reference)
		100-199	1.11 (0.97, 1.28)	1.57 (0.65, 3.81)	1.02 (0.91, 1.14)	1.03 (0.58, 1.82)
		50-99	1.38 (1.19, 1.59)	1.61 (0.57, 4.55)	1.13 (1.02, 1.26)	1.58 (0.96, 2.61)
		<50	1.72 (1.46, 2.03)	2.28 (0.84, 6.18)	1.34 (1.19, 1.50)	2.06 (1.22, 3.51)
	Recipients of National Basic Living Security Act	None	1.00 (reference)	1.00 (reference)	1.00 (reference)	1.00 (reference)
		Ex- or current recipient	1.03 (0.86, 1.24)	1.83 (1.09, 3.05)	1.09 (0.97, 1.23)	0.78 (0.52, 1.15)
	Economic activity	Yes	1.00 (reference)	1.00 (reference)	1.00 (reference)	1.00 (reference)
		No	1.11 (0.99, 1.23)	1.13 (0.71, 1.78)	1.10 (1.01, 1.19)	0.89 (0.65, 1.21)

Values are presented as adjusted odds ratio (95% confidence interval).

1 Adjusted for age, living alone, marital status, monthly household income, recipients of National Basic Living Security Act, economic activity, frequency of family/neighborhood/friend contact, social activities, stress, depression, experienced being sick in bed all day, accident/intoxication, fall, self-rated health, chewing discomfort, limitations in performing daily activities, pain or discomfort, and unmet health care needs.

## SUPPLEMENTARY MATERIALS

Korean version is available at http://www.e-epih.org/.
