# Supplementary material for: Gender difference in socioeconomic factors affecting suicidal ideation and suicidal attempts among community-dwelling elderly: based on the Korea Community Health Survey
Source: Epidemiol Health. 2020 Jul 13;42:e2020052. doi: 10.4178/epih.e2020052 (PMC7871144; doi:10.4178/epih.e2020052)
Supplement: Supplementary file 1 [file epih-42-e2020052-suppl.docx]

Gender difference in socioeconomic factors affecting suicide ideation and suicide attempts among community-dwelling elderly: based on the Community Health Survey

지역사회 거주 노인의 자살생각 및 자살시도에 미치는 사회-경제적 요인의 성별 차이 : 지역사회건강조사를 활용하여

Jin-Young Jeong

Research Institute of Clinical Epidemiology, Hallym University

국문초록

목적: 본 연구는 지역사회 거주 노인의 자살생각 및 자살시도에 관련된 사회-경제적 요인을 탐색하고자 실시되었다.

방법: 연구대상은 2013년과 2017년 지역사회건강조사에 참여한 65세 이상 129,277명이었다. 자살생각과 자살시도 문항을 활용하여, 비자살생각군(111,344명), 자살생각군(17,487명), 자살시도군(446명)으로 분류하였다. 모든 분석은 성별을 층화하였으며, 관련 요인 분석은 복합표본 로지스틱회귀분석을 실시하였다.

결과: 자살생각 관련 남녀 공통요인은 혼인상태, 친구접촉빈도, 사회활동, 월평균 가구소득이었고, 경제활동은 여성 노인에서만 관련성이 나타났다. 자살시도 관련 요인은 남성 노인은 국민기초생활수급가구, 여성 노인은 연령, 가족접촉빈도, 친구접촉빈도, 그리고 월평균가구소득으로 분석되었다.

결론: 본 연구에서 자살을 생각하게 하는 사회경제적 요인은 남녀 노인이 거의 같았으나, 자살시도로 이어지는 요인은 남녀 노인에서 차이가 있었다. 지역사회 거주 노인의 자살 고위험군 선별 및 자살 예방서비스 개발에 기초자료로 활용되길 기대한다.

Key Words: suicide, socioeconomic factors, aged, community health survey

**Ⅰ. 서론**

우리나라 노인의 자살사망률은 인구 10만 명당 2018년 48.6명으로 2010년 81.9명으로 정점을 찍은 뒤 꾸준히 감소하고 있다[1]. 그러나 자살사망자 4명 중 1명이 노인이며, 전체 자살사망률 26.6명에 비해 1.8배 높은 수준이다.

우리나라는 2018년 노인인구의 비율이 14%를 넘어 본격적인 고령사회로 접어들었다[2]. 고령사회는 의학의 발달, 생활환경의 개선 등에 의해 평균수명이 늘어난 긍정적 결과이지만, 50%에 육박하는 높은 노인 빈곤율[3], 질병을 가진 채 살아가는 기간의 증가, 핵가족화로 인한 가족 지지체계 악화와 같은 부정적 측면도 동반한다.

자살연구의 가장 큰 제한점은 자살사망자를 직접 대상으로 수행할 수 없다는 점이다. 그러므로 자살사망과 가까운 행동일수록, 즉 자살생각 보다는 자살시도 연구가 자살의 원인에 보다 가까이 접근할 수 있다. 하지만 지금까지의 자살시도 대상 연구는 소수의 자살시도자 대상의 질적연구가 주를 이루고 있으며[4,5,6,7], 양적연구는 최근 수행된 소수에 불과하다[8,9,10]. 가장 큰 이유로는, 연구대상자 확보의 어려움 때문으로 생각된다. 대표적 노인조사인 2017년 노인실태조사[11]도 자살시도자는 참여자의 0.9%(89명)로, 자살시도 관련 양적 연구 수행에는 한계가 있다. 고위험군을 선별하고 중재를 제공하며, 모니터링에 기초할 일반화된 결과를 위해서는 일반인구집단 대상의 규모 있는 양적연구가 필요하다.

노인의 삶의 질에 사회경제적 요인이 크게 작용하지만, 국내 선행 노인자살연구에서 인구-사회적, 경제적 요인은 연구의 핵심 주제보다는 통제변수로 이용되고 있다[12,13,14]. 또한 자살생각률은 여자가, 자살시도율과 자살률은 남자가 높은 것으로 보고되고 있으며[1,11,15], 이는 관련요인에 있어서도 성별 간 차이가 있을 수 있음을 시사한다.

이에, 본 연구는 인구집단 대상의 대표성을 가진 대규모 역학조사 자료인 지역사회건강조사(Community health survey, CHS)를 활용하여, 지역거주 노인의 자살행동 관련요인을 성별로 층화하여 파악하고자 하였다. 구체적 연구목적은 첫째, 성, 연령군별 자살생각률과 자살시도율을 산출하였다. 둘째, 신체적-정신적 건강 요인을 통제한 상태에서 인구학적 요인, 경제적 요인 및 사회적 관계와 자살생각 및 자살시도의 관련성을 분석하였다.

**Ⅱ. 연구방법**

1. 연구대상자

CHS에서 자살문항 조사는 4년 순환조사로 실시되며, 현재까지 총 3차례(2009년, 2013년, 2017년)에 걸쳐 전국공통문항으로 조사되었으며, 본 연구에서는 2013년과 2017년 CHS 데이터를 사용하였다. 노인 자살률은 2010년을 기점으로 감소 추세로 전환되었으며, 자살률 상승 시기인 2009년도는 관련 요인도 이후 조사와 상이할 가능성이 있다고 판단, 분석에 포함시키지 않았다.

본 연구의 대상자인 65세 이상 참여자 중 자살생각 및 자살시도 무응답자 65명과 자살생각에는 ‘아니오’이면서 자살시도에 ‘예’로 응답한 45명을 제외한 129,277명 최종 분석대상이 되었다.

2. 자료수집방법

CHS는 지역보건의료계획 수립 및 지역보건사업 성과를 평가할 수 있는 시군구 단위 건강통계 산출을 목적으로 2008년에 도입되었다[16]. 목표모집단은 국내 거주하는 만 19세 이상 모든 성인으로 시군구당 900여 명(목표오차 ±3%)을 표본으로 선정, 매년 전국 약 23만 명을 조사한다[17]. 표본추출은 주민등록주소자료를 추출틀로 하여, 확률비례계통추출법으로 1차 표본지점, 계통추출법으로 2차 표본가구를 선정하여 추출한다. 조사원이 가구를 방문하여 노트북에 탑재된 전자조사표(Computer Assisted Personal Interviewing, CAPI)를 이용하여 조사한다. 조사내용은 세대유형, 가구소득, 건강행태, 예방접종 및 검진, 이환, 의료이용, 사고 및 중독, 활동제한 및 삶의 질, 보건기관 이용, 사회 물리적 환경, 개인위생, 여성건강, 교육 및 경제활동 영역을 포함한다. CHS는 질병관리본부 생명윤리위원회의 승인을 받았다. 2013년 승인번호는 2013-06EXP-01-3C이며, 2017년 이후에는 생명윤리 및 안전에 관한 법률 시행규칙 제2조 2항에 근거하여 인간대상연구에 해당되지 않음으로 심의대상에서 제외되었다. 원시자료는 CHS 홈페이지에서 신청(2020년 1월 19일)하여 담당자 승인을 받은 후 다운로드 하였다.

**3. 분석변수**

1) 종속변수

본 연구의 종속변수는 자살생각과 자살시도이며, 각각 단일문항으로 구성되어 있다. 자살생각은 ‘최근 1년 동안 죽고 싶다는 생각을 해 본 적이 있습니까?’, 자살시도는 ‘최근 1년 동안 실제로 자살시도를 해 본 적이 있습니까?’라는 질문에 ‘예’ 또는 ‘아니오’에 응답하도록 되어있다. 본 연구의 목적이 자살생각과 자살시도에 미치는 요인을 탐색하는 것이므로, 자살생각 및 자살시도의 응답결과를 바탕으로 자살생각을 한 적이 없는 군(no suicide ideation, 111,344명), ② 자살시도 경험이 없는 자살생각군(suicide ideation only, 17,487명)과 ③ 자살시도군(suicide attempt, 446명)으로 분류하였다.

2) 독립변수

독립변수에는 인구학적 요인(연령, 혼인상태, 독거여부), 경제 수준(월평균가구소득, 국민기초생활수급가구, 경제활동), 사회적 네트워크(가족/친구/이웃과의 접촉 빈도, 사회활동) 변수를 포함시켰다.

연령은 65-74세, 75세 이상으로, 혼인상태는 ‘배우자가 있으며 동거 중(유배우자)’, ‘사별’, ‘별거/이혼/미혼’으로 분류하였다. 독거여부는 세대유형을 활용하여 1인가구를 ‘독거’로 분류하였다. 월평균가구소득은 2013년에는 개방형으로, 2017년에는 폐쇄형으로 조사되었다. 본 연구에서는 4군(50만원 미만, 50-99만원, 100-199만원, 200만원 이상)으로 범주화 하였다. 국민기초생활수급가구는 ‘현재 또는 과거 수급가구’와 ‘아니다’로 범주화 하였다. 사회적 네크워크 중 친척(가족포함), 이웃, 친구 접촉빈도는 ‘월 1번 미만’, ‘월 1-3번’, ‘주 1번 이상’으로 범주화 하였다. 사회활동은 종교활동, 친목활동, 여가/레저 활동, 자선단체 활동 중 하나 이상에 월 1회 이상 정기적으로 참여하는 것으로 정의하였다.

3) 통제변수

통제변수로는 자살생각 및 자살시도에 영향을 미치는 것으로 알려진 정신건강(우울감, 스트레스), 신체적 기능(낙상, 침상와병, 사고・중독, 일상활동 제한), 삶의 질(주관적 건강수준, 저작불편감, 통증・불편감), 의료이용(필요의료서비스 미치료 경험) 변수를 포함하였다.

**4. 자료분석**

CHS는 모집단인 시-군-구 단위 표본설계를 통해 추출한 표본을 조사하며, 표본설계구조를 바탕으로 가중치를 부여한다[17]. 본 연구의 모든 분석은 CHS의 연구설계를 고려하여 복합표본분석을 실시하였다.

연구대상자의 변수별 분포(Table 1)는 명(%)로 제시하였으며, 가중치를 적용한 %(표본오차)를 함께 제시하였다. Table 2는 비자살생각군, 자살시도 경험이 없는 자살생각군과 자살시도군의 인구 사회학적 특성을 교차분석을 통해 비교하였다.

자살생각 및 자살시도 관련요인 분석은 로지스틱회귀분석을 사용하였다(Table 3). 자살생각 관련요인은 자살생각을 한 적이 없는 군과 자살시도 경험이 없는 자살생각군을 분석대상으로, 자살시도 관련요인은 자살시도 경험이 없는 자살생각군와 자살시도군을 분석대상으로 정의하였다. 다변량 로지스틱회귀분석 시 정신건강(우울, 스트레스), 신체기능(낙상, 침상와병, 사고・중독, 일상활동 제한), 삶의 질(주관적 건강수준, 저작불편감, 통증・불편감), 그리고 필요의료서비스 미치료경험을 보정하였다. 모든 분석은 성별 비교를 위해 성별로 층화하였다. 통계분석은 SAS ver 9.4를 사용하였고, 유의수준은 0.05로 정의하였다.

**Ⅲ. 연구결과**

전체대상자는 129,277명이며, 이 중 남자는 41.2%, 여자는 58.8%이었다(Table 1). 75세 이상은 남 39.7%, 여 46.2%로 여성이 더 고령화 되어 있었다. 유배우자는 남 87.2%, 여 45.4%, 독거노인은 남 10.2%, 여 34.5%였다. 남녀 모두 이웃, 가족(친척), 친구 순으로 접촉빈도가 높았다. 월평균가구소득 100만원 미만은 남 43.0%, 여 56.5%, 현재 또는 과거 국민기초생활수급가구는 남 5.5%, 여 8.5%로 여성 노인이 더 빈곤하였다.

자살생각률은 여자(15.7%)가 남자(9.9%)보다 1.6배 높았다(Figure 1). 남녀 모두 연령과 함께 자살생각률이 증가하였으며 연령군별 성별 격차비(여자/남자)는 65-74세 1.61배. 75-84세 1.54배, 85세 이상 1.68배로 유사하였다. 자살시도율은 남자(3.5%)가 여자(2.7%)보다 1.3배 높았으며, 남자는 85세 이후, 여자는 75세 이후 자살시도율이 감소하였다.

자살생각군과 자살시도군의 인구사회학적 특성 비교는 table 2에 제시하였다. 남자는 연령을, 여자는 사회활동과 경제활동을 제외하고 모든 요인에서 두 군간 차이가 있었다. 먼저 연령을 살펴보면, 남자는 65-74세, 75세 이상의 비율이 두 군 모두 6:4 정도인 반면, 여자는 자살생각군 5:5, 자살시도군 7:3 정도로 군간 차이가 두드러졌다. 혼인상태는 남녀 모두 배우자와 동거는 자살생각군이 이혼/미혼/별거는 자살시도군이 높았다. 독거 또한 남녀 모두 자살시도군이 높았다. 사회적 네트워크 요인을 살펴보면, 가족(친척), 이웃, 친구와 주 1회 이상 접촉, 사회활동 또한 자살시도군이 낮아 자살시도군의 네트워크가 더 빈약한 것으로 나타났다. 경제적 요인 중 월평균가구소득 50만원 미만의 분율, 현재 또는 과거 국민기초생활수급가구 모두 자살시도군이 높았다. Table 2의 p-값은 자살생각군과 자살시도군 간 차이에 대한 p-값이다.

자살생각 및 자살시도 관련요인 분석에는 통제변수를 보정하였으며, 결과는 table 3에 제시하였다. 남자의 자살생각 관련요인은 혼인상태, 월평균가구소득, 친구접촉빈도, 사회활동이었으며, 자살시도 관련요인은 국민기초생활수급가구로 나타났다. 여자의 자살생각 관련요인은 혼인상태, 월평균가구소득, 경제활동, 친구접촉빈도, 사회활동였으며, 자살시도 관련요인은 연령, 월평균가구소득, 가족(친척)접촉빈도와 친구접촉빈도로 나타났다.

**Ⅳ. 고찰 및 결론**

본 연구에서 남성은 자살생각과 자살시도 모두 연령에 따른 특이성이 발견되지 않은 반면, 여성의 경우 65-74세 대비 75세 이상 노인의 자살시도 위험이 낮았다. 한국복지패널(The Korean Welfare Panel study) 6차년도 데이터를 활용한 자살관련 요인을 탐색한 Doh Moonhak과 Hoe Maanse(2015)는 낮은 연령, 높은 우울, 독거, 낮은 가족관계 만족도를 자살시도 요인으로 보고했다[18]. 낮은 연령에서 자살시도가 높은 이유는 첫째, 새롭게 노인인구로 유입되는 젊은 노인의 자살위험이 과거 대비 높아지고 있으며, 둘째, 고령일수록 자살에 대한 표현을 꺼리기 때문[19]에 75세 이상 노인들이 자살시도에 대한 보고가 실제보다 낮게 측정되었을 가능성이 있다고 설명했다.

본 연구에서 자살시도율과 달리 자살사망률은 연령과 함께 높아진다. 2018년 노인의 인구 10만 명당 65-74세, 75-84세, 85세이상 자살사망률은 각각 남자 58.8명, 117.4명, 140.9명, 여자 16.0명, 28.5명, 42.6명이었다. 남녀 모두 고령일수록 자살시도율은 낮고 자살사망률은 높다. 이런 결과는 고령의 높은 자살성공률로 설명할 수 있을 것이다. 젊은 연령에 비해 노인은 자살의도를 잘 알리지 않고 보다 치명적인 수단을 사용하며, 결과적으로 자살성공률이 높다고 알려져 있다[20]. 또 한가지는, 자살시도율(남 3.5%, 여 2.7%)은 성별 차이가 크지 않은데 비해 자살사망률은 남자가 여자 보다 3.5배 높다. 이는, 여자 보다는 남자의 자살성공률이 높을 가능성을 시사한다. 2018년 자살실태조사에 따르면 남자는 여자보다 자살 준비 정도 및 실패하지 않기 위한 계획이 구체적이며, 자살로 이어지는 비율이 높았다[21]. 그러므로 비록 자살시도율은 낮지만 초고령 노인은 자살성공률이 높은 고위험군이므로 주의 깊은 관심이 필요하다고 할 수 있다. 자살시도는 추가적인 자살시도의 강력한 예측요인이며 반복적인 자살시도는 결국 자살사망으로 이어질 가능성을 높인다. 그러므로 여성에 비해 높은 자살시도율을 보인 남성과 연소노인에 대한 관심 또한 매우 중요하다.

노인의 혼인상태와 자살시도 관련성에 대해서는 연구마다 상이한 결과를 보여준다. 결혼이 자살시도를 예방한다는 연구[22,23]와 관련이 없다는 연구[10,24]도 있다. 그러나 이 연구들은 성별을 층화하지 않았기에 본 연구와 직접 비교는 어렵다. 배우자와의 사별은 인생에서 겪는 매우 부정적 사건 중 하나이다. 남성 노인의 사별(8.7%)은 여성 노인(52.1%)보다 발생 가능성이 크게 낮다(Table 1). 그러므로 사별에 의한 충격과 상실감이 여성 노인보다 더 크고 그로 인해 자살생각 위험도 더 클 것으로 판단된다. 더불어 남성 노인의 가사, 요리 등 독립적인 생활에 필요한 기술의 부족도 자살생각을 높이는데 기여했을 수 있다. 그러나 배우자의 사별은 남녀 노인 모두 자살시도에까지 영향을 주지는 않았다. 노인의 54.0%가 2개 이상의 만성질환을 보유하고 있으며[12], 노인의 5대 사망원인(암, 심장질환, 뇌혈관질환, 폐렴, 당뇨병)이 전체 사망의 57.2% 를 차지한다[1]. 노년기 사별이 질병진단-치료-악화-사망의 과정을 거쳐 발생한 사건이라면, 그 과정 중 어느 정도의 심리적 준비가 되었기에 상실감으로 인한 자살생각은 높으나 자살시도로까지는 이어지지 않았을 수 있다.

또 한가지는, 사회적 네트워크의 완충작용 가능성이다. 사회적지지체계는 자살시도의 보호요인으로 알려져 있다[10,25-28]. 배우자를 비롯 동거가족은 신체적 기능에 제한이 있는 노인의 일상생활을 용이하게 할 뿐 아니라 정신적 지지를 제공, 정서적 악화를 막아 자살행동을 예방할 수 있을 것이다.

본 연구에서 정서적 지지 부족에 의한 자살시도는 여성 노인에서 유의미했다. 가족(친척)과 월 1회 미만 접촉 및 친구와 월 1-3회 접촉하는 경우 자살시도 위험이 높았다. 가족은 정서적지지뿐 아니라 경제적 지지를 함께 제공하는 관계이기에 가족과의 접촉빈도는 자살시도를 예측하는 핵심 척도가 될 수 있다. 친구 접촉빈도는 월 1-3회인 경우 자살시도 위험이 높았으나, 이보다 접촉빈도가 낮은 월 1회 미만은 그렇지 않았다. 월 1회 미만 연락하는 친구는 친밀도가 낮은 관계로 판단되며, 그런 관계에게는 정서적 지지를 기대하지 않기 때문일 수 있다. 선행연구에서도 가깝게 지내거나 도움을 주는 사람이 많을수록[13], 친목활동을 할수록[10] 자살시도 위험이 낮았다.

종합하면, 가장 가까운 지지체계가 무너지면 자살생각 위험이 높아지며, 완충작용을 하는 그 외곽의 지지체계까지 무너지면 자살시도 위험이 높아지는 것으로 해석된다. 본 연구에서는 혼인상태와 개인적 네트워크의 접촉빈도로만 사회적 지지를 측정, 지지체계의 견고함은 파악하지 않았다. 지지체계의 견고성 평가는 관계의 친밀도, 접촉의 양적 질적 수준에 대한 측정이 함께 수행되어야 할 것이다. 또한 1인 가구의 증가, 가족관계의 변화 등으로 인해 노인부양에 대한 공적 책임 강화가 요구되고 있으므로, 사회복지사, 방문간호사 등 공적 네트워크의 기여도에 대한 평가도 필요하다.

경제적 요인 중 자살시도 요인은 남자는 ‘과거 또는 현재 국민기초생활수급가구’인 반면 여자는 ‘100만원 미만의 월평균가구소득’으로 나타났다. 이는 경제적 요인이라는 공통점은 있지만 남자는 상대적 빈곤, 여자는 절대적 빈곤이라는 점에서 차이가 있다.

국민기초생활보장법(National Basic Living Security Act)에 의한 2019년 현재 수급자는 전체 인구의 3.6%(1,881,357명)이다[29]. 남자의 경우 성 역할이 분명한 전통적인 가족 구조에서 경제적 주체의 역할을 수행해 왔다. 국민기초생활수급 대상이 되었다는 것은 곧 최빈가구임을 증명하는 것이며, 이는 자신의 경제적 능력 상실이 그 원인으로 스스로를 더 이상 무가치한 존재로 평가하게 될 가능성이 높다. 남성 노인의 자살생각 예측요인을 연령군에 따라 분석한 연구에서, 기초생활보장 수급여부는 전기노인(65-74세)에서 유의성을 보였다[30]. 그에 비해 여성 노인은 경제적 자립의 책무에 대한 기본인식이 남성 노인과는 차이가 있으며, 이에 월평균가구소득이라는 절대적 경제수준이 자살시도에 영향을 미치는 것으로 생각된다.

노인의 상대적 빈곤율(경상소득 기준 중위소득 50% 이하)은 2018년 2/4분기 49.4%로, 노인 2명 중 1명은 빈곤한 상태이다[3]. 노인의 빈곤 개선을 위해 2014년 7월 도입된 기초연금은 노인인구의 대부분인 70%에게 지급, 보편적 소득보장제도의 성격을 띤다. 따라서 공적부조에 부정적 인식을 가진 남성 노인도 기초연금 수급에 대해서는 부정적인 인식이 낮을 것으로 판단된다. 기초연금 도입은 전반적으로 소득증진과 소득불평등을 완화하고 더불어 심리적 효과도 있는 것으로 평가된다[15]. 이에 기초연금의 지급 및 증액은 노인자살 감소에 기여할 것으로 기대되나, 기존 소득이 아주 적은 경우 기초연금을 수급하더라도 빈곤선 탈피에는 한계가 있다. 그러므로 노인일자리 창출, 기초연금 증액과 같은 소득정책과 더불어 경제적 어려움에 기인한 자살행동을 완충해 줄 정서적 지지 강화에도 노력을 기울여야 할 것이다. 지역사회 공동체의 사회적 자원이 개인의 열악한 사회적지지 자원을 대체할 수 있을 것으로 기대한다.

본 연구는 다음과 같은 제한점을 가진다. 첫째, 본 연구는 단면조사 연구로 노인의 인구-사회적 특성과 자살시도간의 인과관계를 밝힐 수는 없다. 둘째, 본 연구는 비교적 치명도가 낮은 자살시도 방법을 사용하고 현재 해당 지역사회에서 비교적 건강하게 생활하는 노인들이 선택적으로 연구에 포함되었을 가능성이 있다. 셋째, 자살은 자살생각, 자살계획, 자살시도의 과정을 거치는 것으로 알려져 있지만, CHS는 자살계획에 대한 정보가 수집되지 않아 자살계획 관련 요인은 분석하지 못했다. 또한 자살생각 문항은 ‘죽고 싶다는 생각을 해본 적이 있는지’에 대한 응답이므로 실제 ‘자살생각’을 반영하지 못했을 가능성이 있다. 더불어 본 연구에서 사용된 자살시도 설문에는 자살시도 방법, 횟수, 치명도 수준 등이 포함되지 않아 보다 심층적인 분석을 수행할 수 없었다. 넷째, 본 연구는 충분한 자살시도자 수 확보를 위해 두 개 년도의 데이터를 병합하였다. 그럼에도 자살시도자는 446명(남 183, 여 263)에 불과하다. 이를 성별로 층화하고 다변량분석을 실시함으로써 해당자가 적은 일부 변수에 대해서는 충분한 통계적 검정력을 확보하지 못했을 수 있다. 다섯째, 2013년과 2017년도의 자살생각 및 자살시도 관련요인이 다르다면 2개년도 데이터를 합침으로써 관련성의 크기가 희석되었을 가능성도 있다. 특히, 기초연금제도는 2014년 도입 되었으므로 2013년과 2017년의 노인의 경제적 특성에 있어서는 차이가 있을 가능성이 높다. 이에 기초연금수급이 노인, 특히 빈곤층 노인의 자살시도에 미치는 영향에 대한 후속연구가 이루어질 필요가 있다.

본 연구는 인구-사회학적 특성과 자살시도와의 관련성에 대해 분석하였고, 연령군, 경제적 요인, 사회적 네트워크 종류에 따른 성별 차이가 있음을 확인하였다. 본 연구결과를 바탕으로 경제적으로 빈곤한 노인, 사회적 지지기반이 빈약한 노인을 자살시도 고위험군으로 선별하고, 보다 체계적으로 추적-관찰할 것을 제안한다.

Acknowledgements

이 논문은 2012년 정부(교육과학기술부)의 재원으로 한국연구재단의 지원을 받아 수행된 연구임(NRF-2012S1A6A3A01033504).

**참고문헌**

1. Statistics Korea. 2018 Datasets and Related Documentation for Mortality Data [cited 2020 May 9]. Available from: http://kostat.go.kr/portal/korea/index.action (Korean).

2. Statistics Korea. Resident registration population. administrative district (si-gun –gu), gender demographics [cited 2020 May 9]. Available from: http://kosis.kr/ statisticsList/statisticsListIndex.do?menuId=M_01_01&vwcd=MT_ZTITLE&parmTabId=M_01_01#SelectStatsBoxDiv (Korean, author’s translation).

3. Korea Institute for Health and Social Affairs. Statistical Yearbook of Poverty 2018 [cited 2020 May 15]. Available from: https://www.kihasa.re.kr/web/publicati on/newbooks_pdsissue/view.do?menuId=46&tid=71&bid=200&ano=1504 (Korean).

4. Im Mi Young, Kim Yun Jeong. A Phenomenological Study of Suicide Attempts in Elders. Journal of Korean Academy of Nursing 2011;41(1):61-71 (Korean).

5. Kim Yu Jin. A Study on Experiences of Older Adults' Getting Back to Life and Rediscovering a 'Will to Live' Following Suicide Attempts. Korean Journal of Social Welfare 2013;65(1):127-146 (Korean).

6. Gwang-Sook Lee, Gyun Choi. Phenomenological Study on the Experiences of Latter-aged Elderly Suicide Attempt. Journal of the Korea contents association 2015;15(7):244-254 (Korean).

7. Oh Jung A, Shim Uibo, Han Kyuryang. Study of the Life History of the Elderly with Suicide Attempt Experience. Journal of Social Science 2018;29(1):.3-21 (Korean).

8. Oh-Gyun Kwon. A study on the determinants of suicide attempts among the elderly living alone. Journal of Korean Home Management Assessment 2014;32(5):207-219 (Korean).

9. Junsoo Ro, Jongheon Park, Jinsuk Lee, Hyemin Jung. Factors That Affect Suicidal Attempt Risk Among Korean Elderly Adults: A Path Analysis. J Prev Med Public Health 2015;48:28-37.

10. Chang SH, Suh EY, Choi HJ. Risk Factors on Suicidal Ideation and Suicidal Attempt among Community dwelling Older Adults: Based on 2014 Community Health Survey. Korean J Rehabil Nurs 2017;20(2):111-121 (Korean).

11. Ministry of Health and Welfare, Korea Institute for Health and Social Affairs. 2017 The Survey of Living Conditions and Welfare Needs of Korean Older Persons [cited 2020 May 01]. Available from: http://www.prism.go.kr/homepage /entire/retrieveEntireDetail.do?pageIndex=1&research_id=1351000-201700250&leftMenuLevel=160&cond_research_name=%EB%85%B8%EC%9D%B8%EC%8B%A4%ED%83%9C%EC%A1%B0%EC%82%AC&cond_research_start_date=&cond_research_end_date=&pageUnit=10&cond_order=3 (Korean)

12 Jeong Jin-Young, Lee Sooin. Physical Health Related Factors on Suicidal Thought among Community-dwelling Elderly in Korea. Korean Public Health Research 2017;43(4):59-72 (Korean).

13. Jung Myounghee, Heo Seonghui. Study on the Effects of Suicide Loss Experience on Suicidal Ideation, Plan and Attempt of the Elderly. Korean Journal of Gerontological Social Welfare 2017;72(1):305-333 (Korean).

14. Hwang Jung Woo, Lee Kang Uk, Kim Jung Yoo, Lee Dong Ha, Kim Doo Myung. The affection of depression of the elderly living together and the elderly living alone on suicidal ideation and the moderating effects of personal relation. Mental Health & Social Work 2017;45(1):36-62 (Korean).

15. Jin-Young Jeong. Economic factors and suicidal ideation among metropolitan and rural dwelling elderly in Korea: based on the 2009, 2013, 2017 Community Health Survey. Korean Public Health Research 2019;45(3):117-129 (Korean).

16. Korea Centers for Disease Control and Prevention. Community health survey [cited 2020 Apr 30]. Available from: https://chs.cdc.go.kr/chs/bsnsIntrcn/bsns SumryMain.do (Korean).

17. Ministry of Health and Welfare, Korea Centers for Disease Control and Prevention. 2008-2018 Community health statistics at a glance [cited Apr 27]. Available from: https://chs.cdc.go.kr/chs/stats/statsMain.do (Korean).

18. Doh Moonhak, Hoe Maanse. An Exploratory Study Of Ecological System Factors Influencing Elderly Suicide: ROC Curve Analysis. Social Science Research Review 2015;31(2):45-72 (Korean).

19. Duberstein PR, Conwell Y, Seiditz L, Lyness JM, Cox C, Caine ED. Age and suicidal ideation in older depressed inpatients.” American Journal of Geriatric Psychiatry 1999;7:289-296.

20. Ministry of Health, Welfare and Family Affairs, Korea Centers for Disease Control and Prevention. The main cause of suicide attempts by elderly is health problems-Older people who are physically and mentally ill, have a greater risk of suicide. KCDC press. 2007 [cited 2020 May 4]. Available from: http://www.welfare24.net/ab-3780-212?PB_1384961453=1&PB_1385010203=1&PB_1383383930=1&PB_1383673525=1&PB_1383673596=1&PB_1383673656=1&PB_1383670495=9&PB_1516623282=4 (Korean, author’s translation).

21. Ministry of Health and Welfare. 2018 National Survey on Suicide [cited 2020 May 2]. Available from: http://www.prism.go.kr/homepage/entire/retrieveEntire Detail.do;jsessionid=54F384B464EED4354A8C5F898F7FE56A.node02?cond_research_name=&cond_research_start_date=&cond_research_end_date=&research_id=1351000-201900363&pageIndex=223&leftMenuLevel=160 (Korean).

22. Qin P, Agerbo E, Mortensen PB. Suicide risk in relation to family history of completed suicide and psychiatric disorders. A nested case-control study. Ugeskr. Laeger 2003;165(25):2573–2577.

23. Stefan Wiktorsson, Bo Runeson, Ingmar Skoog, Svante Ostling, Margda Waern. Attempted Suicide in the Elderly: Characteristics of Suicide Attempters 70 Years and Older and a General Population Comparison Group. Am J Geriatr Psychiatry 2010;18(1):547-67.

24. Wei Zhang, Hansheng Ding, Peng Su, Guangfeng Duan, Rong Chen, Junrui Long, Lixia Du, Chunyan Xie, Chunlin Jin, Chaoqun Hu, Zixue Sun, Lingling Gong and Wenhua Tian. Does disability predict attempted suicide in the elderly? A community-based study of elderly residents in Shanghai, China. Aging & Mental Health 2016;20(1):81-87.

25. Duberstein PR, Conwell Y, Conner KR, Eberly S, Evinger JS, Caine ED. Poor social integration and suicide: Fact or artifact? A case-control study. Psychological Medicine 2004;34(7):1331-1337.

26. Beautrais AL. A case control study of suicide and attempted suicide in older adults. Suicide Life Threat Behav 2002;32(1):1–9.

27. Turvey CL, Conwell Y, Jones MP. Risk factors for late-life suicide: a prospective, community based study. Am J Geriatr Psychiatry 2002;10(4):398–406.

28. Tsai AC, Lucas M, Kawachi I. Association Between Social Integration and Suicide Among Women in the United States. JAMA Psychiatry 2015;72(10):987-993.

29. Ministry of Health and Welfare. Bokjiro Social security statistics. Statistics on the number of households and recipients by basic livelihood security recipient classification [cited 2020 May 15]. Available from: https://www.bokjiro.go.kr /nwel/welfareinfo/sociguastat/retrieveSociGuaStatList.do (Korean).

30. Lee Si Eun, Hong Gwi-Ryung Son. Predictors of suicidal ideation in community-dwelling older men: a comparison between young-old and old-old. J Korean Acad Psychiatr Ment Health Nurs 2016;25(3):217-226 (Korean).
